# Serum dehydroepiandrosterone levels are associated with lower risk of type 2 diabetes: the Rotterdam Study

**DOI:** 10.1007/s00125-016-4136-8

**Published:** 2016-10-22

**Authors:** Adela Brahimaj, Taulant Muka, Maryam Kavousi, Joop S. E. Laven, Abbas Dehghan, Oscar H. Franco

**Affiliations:** 1 Department of Epidemiology, Erasmus University Medical Center, PO Box 2040, 3000 CA Rotterdam, the Netherlands; 2 Division of Reproductive Medicine, Department of Obstetrics and Gynaecology, Erasmus MC, Rotterdam, the Netherlands

**Keywords:** Androstenedione, DHEA, DHEAS, Food supplement, Independent marker, Type 2 diabetes

## Abstract

**Aims/hypothesis:**

Previous literature documents controversial results for the impact of dehydroepiandrosterone (DHEA) in glucose metabolism. We aimed to assess the associations between serum levels of DHEA and its main derivatives DHEA sulphate (DHEAS) and androstenedione, as well as the ratio of DHEAS to DHEA, and risk of type 2 diabetes.

**Methods:**

We used data on serum levels of DHEA, DHEAS and androstenedione from 5189 middle-aged and elderly men and women from the prospective population-based Rotterdam Study. Type 2 diabetes was defined as a fasting blood glucose ≥7.0 mmol/l or a non-fasting blood glucose ≥11.1 mmol/l.

**Results:**

During a median follow-up of 10.9 years, 643 patients with incident type 2 diabetes were identified. After adjusting for age, sex, cohort, fasting status, fasting glucose and insulin, and BMI, both serum DHEA levels (per 1 unit natural log-transformed, HR 0.76, 95% CI 0.67, 0.87) and serum DHEAS levels (per 1 unit natural log-transformed, HR 0.82, 95% CI 0.73, 0.92) were inversely associated with risk of type 2 diabetes in the total population. Further adjustment for alcohol, smoking, physical activity, prevalent cardiovascular disease, serum total cholesterol, use of lipid-lowering medications, systolic BP, treatment for hypertension, C-reactive protein, oestradiol and testosterone did not substantially affect the association between DHEA and incident type 2 diabetes (per 1 unit natural log-transformed, HR 0.80, 95% CI 0.65, 0.99), but abolished the association between DHEAS and type 2 diabetes. Androstenedione was not associated with risk of type 2 diabetes, nor was DHEAS to DHEA ratio.

**Conclusions/interpretation:**

DHEA serum levels might be an independent marker of type 2 diabetes.

**Electronic supplementary material:**

The online version of this article (doi:10.1007/s00125-016-4136-8) contains peer-reviewed but unedited supplementary material, which is available to authorised users.

## Introduction

Dehydroepiandrosterone (DHEA), mostly present as its sulphated ester (DHEAS), is the most abundant circulating adrenal steroid hormone in healthy adults. In both men and women, peak serum levels of DHEA and DHEAS occur around 25 years of age, with levels declining steadily from the third decade onwards [1]. Similarly, both glucose tolerance and insulin sensitivity decrease with ageing [2].

DHEA is available as a health food supplement in the USA, but previous literature on the effects of supplemental DHEA on glucose metabolism in healthy humans is controversial [3–6]. Evidence from animal studies indicates that DHEA treatment could result in increased insulin-induced glucose uptake in rat models of type 2 diabetes and moderate the severity of diabetes [7, 8]. Along the same lines, a randomised controlled trial (RCT) of elderly women and men with an age-related decrease in DHEA showed that DHEA replacement reduced abdominal fat and improved insulin sensitivity [9]. Further, but not all, RCTs have reported positive effects of DHEA supplementation on reducing body fat and improving glucose metabolism [10–14]. In observational studies, levels of DHEAS have also been associated with lower BMI and visceral fat in women [15], and plasma glucose and serum insulin concentrations in non-diabetic men [16].

Despite extensive research on DHEA and insulin action, information about the role of DHEA in type 2 diabetes risk remains scarce. Studies prospectively investigating the association between DHEA or DHEAS and type 2 diabetes are limited and have been conducted mainly in women [17, 18]. Sex differences have been suggested, and it remains unclear whether the effects of DHEA on risk of type 2 diabetes are different in women and men [11–14, 19, 20]. We therefore aimed to prospectively examine the associations between DHEA and its main derivatives DHEAS and androstenedione, as well as the ratio of DHEAS to DHEA, and incident type 2 diabetes in healthy middle-aged and elderly men and women.

## Materials and methods

### Study population

The Rotterdam Study is a population-based cohort study of individuals aged 45 years and over who live in the Ommoord district of Rotterdam, the Netherlands. The rationale and design of the Rotterdam Study is described elsewhere [21]. In brief, all inhabitants of the Ommoord district aged 55 years or older were invited to participate (*n* = 10,215). At baseline (1990–1993), 7983 participants were included (RS-I). In 2000, an additional 3011 participants were enrolled (RS-II), consisting of all persons living in the study district who had turned 55 years of age. Follow-up visits were held every 3–5 years. For this study, we used measurements of DHEA and its derivatives that were made during the third visit of the first cohort (RSI-3) and the baseline examinations of the second cohort (RSII-1).

The Rotterdam Study has been approved by the Medical Ethics Committee according to the Wet Bevolkingsonderzoek: ERGO (Population Study Act: Rotterdam Study), executed by the Ministry of Health, Welfare and Sports of the Netherlands. All participants gave informed consent for their participation in the study and for information to be obtained separately from treating physicians and pharmacies.

### Population under analysis

The present study used data from the third visit of the first cohort (RSI-3) and the baseline examinations of the second cohort (RSII-1). Overall, 6923 participants were eligible for blood measurements and available for type 2 diabetes follow-up (3923 postmenopausal women and 3000 men). Of these, 937 individuals (483 postmenopausal woman and 454 men) with prevalent diabetes were excluded. Furthermore, for 797 participants (422 postmenopausal women and 375 men), there was no information on DHEA and its derivatives, so they were therefore excluded from the analysis, leaving 5189 participants for analysis (see electronic supplementary material [ESM] Fig. 1). Concentrations of sex steroids were assessed in non-fasting samples from 270 of these participants. A sensitivity analysis was performed excluding this group from the analysis.

### Ascertainment of type 2 diabetes

The participants were followed from the date of the baseline visit to the research centre onwards. At baseline and during follow-up, cases of type 2 diabetes were ascertained through active follow-up using general practitioners’ records, hospital discharge letters and serum glucose measurements from Rotterdam Study visits, which take place approximately every 4 years [22]. Type 2 diabetes was defined, according to current WHO guidelines, as a fasting blood glucose ≥7.0 mmol/l, a non-fasting blood glucose ≥11.1 mmol/l (when fasting samples were absent) or the use of blood glucose-lowering medication [23]. Information regarding the use of blood glucose-lowering medication was derived from both structured home interviews and linkage to pharmacy records [22]. At baseline, more than 95% of the Rotterdam Study population was covered by the pharmacies in the study area. All potential events of type 2 diabetes were independently adjudicated by two study physicians. In cases of disagreement, consensus was sought from an endocrinologist. Follow-up data were complete until 1 January 2012.

### Hormone measurements

Sex hormone-binding globulin (SHBG) was measured using the Immulite 2000XPi platform (Siemens, Los Angeles, CA, USA), while thyroid-stimulating hormone (TSH) was measured on the Vitros Eci (Ortho Diagnostics, Raritan, NJ, USA). Oestradiol was measured using a COBAS 8000 Modular Analyzer (Roche Diagnostics, Rotkreuz, Switzerland). The corresponding interassay CV was <7%.

Androstenedione, testosterone and DHEAS were measured on a Waters XEVO TQ-S system (Waters, Milford, MA, USA) using the CHSMSMS Steroids Kit (Perkin Elmer, Turku, Finland). The interassay CVs of androstenedione, testosterone and DHEAS were <6.5%, <5% and <5.9%, respectively. Although assessment of blood measurements, including sex steroids, was performed on fasting samples, some of the participants came to the research centre in a non-fasting state.

### Covariates

Information on current health status, medical history, medication use and smoking behaviour was obtained at baseline for all the participants. Participants were asked whether they were currently smoking cigarettes, cigars or pipes. Alcohol intake was assessed in grams of ethanol per day. History of cardiovascular disease (CVD) was defined as a history of CHD (myocardial infarction, revascularisation, coronary artery bypass graft surgery or percutaneous coronary intervention) or stroke and was verified from the general practitioner’s medical records. Information regarding the use of hormone replacement therapy was derived from structured home interviews. Parental history of diabetes was collected by trained research assistants during home visits.

BP was measured in the sitting position on the right upper arm with a random-zero sphygmomanometer. Physical height (m) and body weight (kg) were measured at baseline with the participants standing without shoes and heavy outer garments. BMI was calculated as weight divided by height squared (kg/m^2^). All biochemical variables were assessed using fasting serum. Insulin, glucose, total cholesterol (TC), HDL-cholesterol (HDL-C), triacylglycerol (TG) and C-reactive protein (CRP) were measured using a COBAS 8000 Modular Analyzer (Roche Diagnostics). The corresponding interassay CVs were insulin <8%, glucose <1.4%, lipids <2.1% and CRP <16.9%. LDL-cholesterol (LDL-C) level was estimated indirectly from measurements of TC, and HDL-C and TGs by means of the Friedewald equation [24]. Physical activity was assessed using an adapted version of the Zutphen Physical Activity Questionnaire [25]. Every activity mentioned in the questionnaire was attributed a metabolic equivalent value according to the 2011 codes [26].

### Statistical analysis

Person-years of follow-up were calculated from study entrance (March 1997 to December 1999 for RSI-3, February 2000 to December 2001 for RSII-1) to the date of diagnosis of type 2 diabetes, death or the censor date (date of last contact of the living), whichever occurred first. Follow-up lasted until 1 January 2012.

Cox proportional hazard modelling was used to evaluate whether DHEA, DHEAS, androstenedione, and DHEAS to DHEA ratio were associated with type 2 diabetes. HRs and 95% CIs were reported. The proportional hazard assumption of the Cox model was checked by visual inspection of log minus log plots and by performing a test for heterogeneity of the exposure over time. There was no evidence of violation of the proportionality assumption in any of the models (*p* for time-dependent interaction terms >0.05). To account for age and sex differences in serum levels of DHEA, DHEAS and androstenedione, we used sex hormone levels adjusted for age and sex by using the residual method [27]. All sex hormones variables were assessed in separate models, continuously and in tertiles. To study the relations across increasing tertiles, trend tests were computed by entering the categorical variables as continuous variables in multivariable Cox’s proportional hazard models. To achieve approximately normal distribution, skewed variables (DHEA, DHEAS, androstenedione, testosterone, oestradiol, SHBG, CRP, TSH, glucose and insulin) were natural log-transformed.

In the base model (model 1), we adjusted for age, sex, cohort (I and II), fasting status (fasting sample vs non-fasting sample). Model 2 included all factors in model 1 as well as BMI (continuous), glucose (continuous) and insulin (continuous). Model 3 included all the covariates from model 2 as well as further potential intermediate factors including metabolic risk factors (TC, systolic BP [SBP; continuous], treatment for hypertension [yes vs no] and use of lipid-lowering medications [yes vs no]), lifestyle factors (alcohol intake [continuous], smoking status [current vs former/never] and physical activity), prevalent CVD (yes vs no) and CRP level (continuous). We also controlled for upstream precursor hormones (ESM Fig. 2), which may act as confounders. Thus, DHEA was adjusted in all models that included either DHEAS or androstenedione. Effect modifications of sex hormones by BMI and sex were tested in the final multivariable model in addition to performing stratified analysis.

We also performed several sensitivity analyses: (1) further adjusting for hormones including downstream metabolites that might be casual intermediates, such as oestradiol and testosterone; (2) further adjusting for SHBG; (3) substituting BMI for waist circumference; (4) substituting TC with HDL-C, TGs and LDL-C; (5) further adjusting for parental history of diabetes (6); further adjusting for TSH; (7) further adjusting by excluding individuals with type 2 diabetes within the first 3 years of follow-up (*n* = 99/643 cases); (8) excluding participants with non-fasting samples (*n* = 270); (9) further adjustment for hormone replacement therapy; and (10) further adjustment for free androgen index. A multiple imputation procedure (*n* = 5 imputations) was used to impute the missing data.

Moreover, we compared the effect of DHEA and its derivatives on 10-year risk prediction of type 2 diabetes by studying the discrimination. Discrimination is the ability of a predictive model to assign a higher risk to individuals who will develop an event in 10 years compared with those who will not. We quantified discrimination for both models by calculating the c-statistic difference between the base model and the models that additionally included DHEA or its derivatives [28]. There is not yet a unique established risk prediction model for type 2 diabetes; therefore, as a base model, we used Wilson’s risk score, including age, sex, parental history of diabetes and BMI [29]. To perform this analysis, we used a combination of ‘foreign’ (https://cran.r-project.org/web/packages/foreign/foreign.pdf) and ‘survC1’ (https://cran.r-project.org/web/packages/survC1/survC1.pdf) R packages. All other analyses were done using IBM SPSS Statistics software version 21.0.0.1 (SPSS, Chicago, IL, USA) and R software version 3.0.1 (R Foundation for Statistical Computing, Vienna, Austria). A *p* value <0.05 was considered statistically significant.

## Results

Table 1 summarises the baseline characteristics of 5189 participants who were free from diabetes at baseline, including 3018 postmenopausal women and 2171 men.

**Table 1:** Selected characteristic of study participants from the Rotterdam Study

Variable	Value
Characteristic, *n*	5189
Age (years)	69 ± 8.3
Males, *n* (%)	2171 (41.8)
Fasting status, *n* (%)	4911 (94.6)
Current smokers, *n* (%)	632 (12.2)
Alcohol intake (g/day)	2.1 (11.6)
BMI (kg/m^2^)	26.7 ± 3.8
Waist circumference (cm)	92.7 ± 11.5
Prevalent CVD, *n* (%)	586 (11.3)
Parental history of diabetes, *n* (%)	463 (8.9)
Oestradiol (pmol/l)	62.8 (137.5)
Total testosterone (nmol/l)	1.4 (24.1)
SHBG (nmol/l)	55.5 (93.1)
TSH (mU/l)	1.8 (4.9)
Insulin (pmol/l)	66 (145)
Glucose (mmol/l)	5.5 (1.8)
CRP (nmol/l)	0.0162 (0.0981)
TC (mmol/l)	5.8 ± 0.9
LDL-C (mmol/l)	3.7 ± 0.9
HDL-C (mmol/l)	1.4 ± 0.4
Use of serum lipid-reducing agents, *n* (%)	605 (11.7)
Hormone replacement therapy, *n* (%)	140 (2.7)
Free androgen index	2.5 (31.8)
TGs (mmol/l)	1.4 (0.8)
SBP (mm/Hg)	142 ± 21
Treatment for hypertension, *n* (%)	1087 (20.9)
DHEA (nmol/l)	8.3 (20.6)
DHEAS (nmol/l)	1819.2 (4672.7)
Androstenedione (nmol/l)	2.5 (4.1)

Values are mean ± SD, or median (interquartile range), unless otherwise indicated

### Survival analysis

During a median follow-up time of 10.9 years, 643 incident cases of type 2 diabetes were identified. After adjusting for age, sex, cohort, fasting status, fasting glucose and insulin, and BMI, both serum DHEA levels (per 1 unit natural log-transformed, HR 0.76, 95% CI 0.67, 0.87) and serum DHEAS levels (per 1 unit natural log-transformed, HR 0.82, 95% CI 0.73, 0.92) were inversely associated with risk of type 2 diabetes (Table 2) in the total population. Further adjustment for alcohol intake, smoking status, physical activity, prevalent CVD, serum TC, use of serum lipid-reducing medications, SBP and treatment for hypertension, and CRP concentration did not materially affect the association between DHEA and incident type 2 diabetes (per 1 unit natural log-transformed, HR 0.76, 95% CI 0.67, 0.87), but it abolished the association between DHEAS and type 2 diabetes (Table 2). Androstenedione was not associated with risk of type 2 diabetes, and neither was the DHEAS to DHEA ratio (Table 2).

**Table 2:** Associations of androstenedione, DHEA and DHEAS with risk of type 2 diabetes in postmenopausal women and men in the Rotterdam Study (*n* = 5189)

Variable	Tertile 1	Tertile 2	Tertile 3	Continuous	*p* value	*p* trend
Androstenedione						
Cases	216	230	197			
Model 1, HR, 95% CI	1	1.09 (0.90, 1.31)	0.92 (0.76, 1.12)	0.92 (0.77, 1.09)	0.3	0.4
Model 2, HR, 95% CI	1	1.07 (0.89, 1.29)	0.82 (0.67, 0.99)^*^	0.82 (0.69, 0.97)	1.8 × 10^−2^	4.5 × 10^−2^
Model 3, HR, 95% CI	1	1.19 (0.97, 1.46)	0.98 (0.77, 1.26)	0.98 (0.78, 1.24)^a^	0.9	0.9
DHEA						
Cases	236	206	201			
Model 1, HR, 95% CI	1	0.79 (0.66, 0.96)	0.76 (0.63, 0.92)^*^	0.79 (0.69, 0.89)	3.0 × 10^−4^	5.0 × 10^−3^
Model 2, HR, 95% CI	1	0.84 (0.69, 1.02)	0.73 (0.60, 0.89)^*^	0.76 (0.67, 0.87)	5.6 × 10^−5^	1.0 × 10^−3^
Model 3, HR, 95% CI	1	0.84 (0.69, 1.02)	0.73 (0.60, 0.89)^*^	0.76 (0.67, 0.87)^a^	8.7 × 10^−5^	2.0 × 10^−3^
DHEAS						
Cases	226	203	214			
Model 1, HR, 95% CI	1	0.86 (0.71, 1.04)	0.91 (0.76, 1.09)	0.88 (0.79, 0.99)	3.5 × 10^−2^	0.3
Model 2, HR, 95% CI	1	0.79 (0.65, 0.96)^*^	0.83 (0.69, 1.01)	0.82 (0.73, 0.92)	1.0 × 10^−3^	6.0 × 10^−2^
Model 3, HR, 95% CI	1	0.91 (0.74, 1.12)	1.13 (0.88, 1.45)	0.94 (0.79, 1.12)^a^	0.5	0.3
DHEAS/DHEA						
Cases	198	215	230			
Model 1, HR, 95% CI	1	1.08 (0.89, 1.31)	1.23 (1.02, 1.49)^*^	1.14 (0.96, 1.34)	0.1	3.0 × 10^−2^
Model 2, HR, 95% CI	1	1.11 (0.92, 1.35)	1.15 (0.95, 1.39)	1.03 (0.88, 1.22)	0.6	0.1
Model 3, HR, 95% CI	1	1.11 (0.92, 1.36)	1.15 (0.95, 1.41)	1.03 (0.87, 1.22)^a^	0.7	0.1

Model 1: adjusted for age, sex, cohort, fasting status

Model 2: model 1 + insulin, glucose and BMI

Model 3: model 2 + alcohol intake, smoking status, physical activity, prevalent CVD, serum TC, use of serum lipid-reducing agents, SBP, treatment for hypertension, C-reactive protein and sex hormones adjusted for each other (androstenedione adjusted for DHEA, DHEAS adjusted for DHEA)

^a^No significant interaction between sex and hormone or ratio (*p* > 0.05)

^*^ *p* < 0.05

### Sensitivity analysis

In the sensitivity analysis, none of the associations was affected by the following: (1) further adjusting for hormones including downstream metabolites that might be casual intermediates such as oestradiol and testosterone; (2) further adjustment for SHBG; (3) substituting BMI for waist circumference; (4) substituting TC for HDL-C, TGs and LDL-C; (5) further adjustment for parental history of diabetes; (6) further adjustment for TSH level; (7) exclusion of individuals with type 2 diabetes within the first 3 years of follow-up; (8) excluding participants with non-fasting samples (*n* = 270); (9) further adjustment for hormone replacement therapy; and (10) further adjustment for free androgen index (Table 3). In addition, no significant interactions were found for any of the hormones or their ratio with sex and BMI in the stratification analysis (Table 3).

**Table 3:** Sensitivity analysis of sex hormones and risk of type 2 diabetes in postmenopausal women and men in the Rotterdam Study (*n* = 5189)

Variable	Androstenedione	DHEA	DHEAS	DHEAS/DHEA
Multivariable model^a^	0.99 (0.79, 1.24)	0.76 (0.67, 0.88)^*^	0.94 (0.80, 1.11)	1.03 (0.87, 1.21)
Multivariable model + sex hormones for each other (oestradiol and testosterone included in the model)	1.01 (0.80, 1.27)	0.80 (0.65, 0.99)^*^	0.94 (0.79, 1.10)	Not included
Multivariable model + SHBG	0.96 (0.78, 1.19)	0.77 (0.67, 0.88)^*^	0.89 (0.77, 1.03)	0.96 (0.83, 1.12)
Multivariable model + waist circumference	0.98 (0.78, 1.23)	0.76 (0.67, 0.87)^*^	0.95 (0.80, 1.12)	1.04 (0.88, 1.22)
Multivariable model + HDL-C + TG + LDL-C	0.98 (0.78, 1.24)	0.77 (0.68, 0.88)^*^	0.97 (0.82, 1.14)	1.06 (0.89, 1.25)
Multivariable model + serum TSH	0.97 (0.77, 1.22)	0.76 (0.67, 0.87)^*^	0.96 (0.81, 1.13)	1.05 (0.89, 1.23)
Multivariable model + parental history of diabetes	0.98 (0.79, 1.22)	0.77 (0.67, 0.88)^*^	0.95 (0.82, 1.09)	1.02 (0.88, 1.18)
Multivariable model excluding the first 3 years of follow-up	1.06 (0.83, 1.36)	0.76 (0.66, 0.88)^*^	0.97 (0.81, 1.16)	1.06 (0.89, 1.27)
Multivariable model excluding non-fasting participants	0.96 (0.77, 1.21)	0.76 (0.67, 0.88)^*^	0.91 (0.78, 1.06)	0.98 (0.84, 1.15)
Multivariable model + hormone replacement therapy	0.98 (0.79, 1.23)	0.76 (0.67, 0.87)^*^	0.95 (0.82, 1.11)	1.03 (0.88, 1.19)
Multivariable model + free androgen index	0.95 (0.75, 1.20)	0.76 (0.67, 0.87)^*^	0.90 (0.76, 1.07)	1.01 (0.85, 1.19)
Incident diabetes cases (*n* = 643)^b^				
BMI (kg/m^2^)^a^				
<25 (*n* = 134)^b^	0.75 (0.45, 1.24)^c^	0.81 (0.59, 1.11)^c^	1.21 (0.82, 1.78)^c^	1.29 (0.89, 1.87)^c^
25–29.9 (*n* = 323)^b^	1.20 (0.87, 1.66)^c^	0.80 (0.66, 0.97)^c*^	0.96 (0.77, 1.21)^c^	1.02 (0.81, 1.29)^c^
≥30 (*n* = 186)^b^	0.84 (0.54, 1.30)^c^	0.67 (0.53, 0.86)^c*^	0.75 (0.55, 1.03)^c^	0.88 (0.65, 1.21)^c^
Sex^a^				
Men (270)^b^	1.17 (0.80, 1.71)	0.82 (0.66, 1.03)	1.02 (0.77, 1.35)	1.10 (0.84, 1.44)
Women (373)^b^	0.88 (0.66, 1.17)	0.74 (0.63, 0.88)^*^	0.91 (0.74, 1.12)	0.99 (0.80, 1.22)

Values are + 1 natural log increase

^a^Multivariable model adjusted for variables in model 3 of Table 2

^b^Incident diabetes cases for each BMI stratum or sex, from 643 cases in total

^c^ *p* for interaction >0.05; ^*^ *p* < 0.05

The c-statistic of the Wilson’s base model for 10-year risk of type 2 diabetes was 0.637 (0.609, 0.666). When we added DHEA to the model, the c-statistic improved to 0.638 (0.612, 0.665), with a difference of 0.001 (−0.003, 0.005). DHEA and its derivatives did not improve Wilson’s base model (ESM Table 1).

## Discussion

In this large prospective population-based cohort study, we found that serum level of DHEA was inversely associated with risk of type 2 diabetes, independent of established diabetes risk factors including BMI, fasting glucose, insulin and CRP. We did not find any evidence regarding sex differences in this association. The association also remained significant after including in the model downstream sex steroid metabolites such as testosterone and oestradiol, cited as risk factors for type 2 diabetes [30]. In our study, no independent association was found between DHEAS, androstenedione or DHEAS to DHEA ratio and risk of type 2 diabetes.

A study of 1612 postmenopausal women prospectively examined the association between DHEA and risk of type 2 diabetes and showed no effect [17]. Our study included both men and women, a larger number of incident cases of type 2 diabetes (643 vs116), a longer follow-up ( median 10.9 vs 4.7 years) and a comprehensive assessment of hormones. Another study by Mather et al showed no association between DHEA and incidence of diabetes, but it was conducted in individuals at high risk of diabetes and involved a short follow-up (median 3.0 years) [31]. Similar to our findings, although an inverse association was suggested in a nested case–control study of 718 postmenopausal women, DHEAS was not statistically significantly associated with a lower risk of type 2 diabetes [18]. In addition, a recent study, including 1258 community-dwelling men and women reported no association between DHEAS and type 2 diabetes overall, but after stratification by sex, higher serum DHEAS levels were protective against type 2 diabetes in men, but not in women [19].

Previous studies in animal have documented therapeutic effects of DHEA in mice used as a model of diabetes [7]. Owing to the striking beneficial effects, observed mostly in animals, of DHEA against diabetes, as well as against other chronic conditions such as cancer, obesity and CVD, DHEA is now available as a food supplement in retail stores [32–34]. The aforementioned conditions are mostly observed in ageing and as accompanying the natural steady decline of DHEA from the third decade onward [1, 35].

However, animal data should be translated with caution to human physiology, taking into consideration the contrast between the lower amounts of DHEA in laboratory animals and the distinctly human role of DHEA, which has a pattern of biosynthesis specific to higher primates [36]. Until recently, there has been conflicting evidence in the literature concerning the effect of DHEA on glucose metabolism in healthy humans. Human data have shown that DHEA administration increases [9, 11], has no effect [13, 20] or decreases [37] insulin sensitivity, leaving the role of DHEA in glucose metabolism and development of type 2 diabetes unclear. Moreover, Villareal et al [9] found that DHEA supplementation for 6 months decreased visceral adiposity, whereas in a 2 year trial, Nair et al [20] did not observe any benefits of DHEA.

Our findings on a protective role of DHEA against type 2 diabetes provide epidemiological evidence in agreement with previous claims for positive effects of DHEA in type 2 diabetes (DHEA has previously also been called ‘elixir of youth’). Pertaining to its mechanisms of action, DHEA is a peroxisome proliferator-activated receptor (PPAR) α agonist [30]. Tenenbaum et al found that bezafibrate, a PPARα receptor ligand, reduced the incidence and delayed the onset of type 2 diabetes in patients with impaired fasting glucose levels [38]. DHEA and DHEAS have been shown to be insulin sensitisers [39], whereas there is less evidence that insulin alters DHEA or DHEAS levels [40]. Perrini et al found that DHEA increases glucose uptake in both human and 3 T3-L1 adipocytes by stimulating GLUT4 and GLUT1 translocation to the plasma membrane [41]. Another study of patients with type 2 diabetes concluded that DHEA administration counteracted oxidative imbalance and advanced glycation end-product formation [42]. In addition, in rat models, it has been shown that DHEA improves glucose uptake via activation of protein kinase C and phosphatidylinositol 3-kinase [43]. Another possible mechanism for the prevention of type 2 diabetes by DHEA is an improvement in endothelial function [14], which is implicated in the development of insulin resistance [44].

Previous studies have suggested sex differences for DHEA and its derivatives in type 2 diabetes [11, 12, 19, 45]. DHEA concentrations are approximately twice as high in women, whereas DHEAS levels are higher in men [46]. In our study, we observed the protective effect of DHEA in men and women, slightly more prominent in women than in men, but the interaction with sex was not significant. The HRs showed the same direction in both women and men, but the association was not significant in men. This might be explained by the smaller number of incident cases of type 2 diabetes in men (*n* = 270) compared with women (*n* = 373) in our study.

Very little is known regarding the role of plasma levels of androstenedione in the pathology of type 2 diabetes. O’Reilly et al showed that serum androstenedione level, other than in its role as a precursor of testosterone, is a useful tool for predicting metabolic risk in women with polycystic ovary syndrome [47]. Our study is the first to investigate the association of serum androstenedione levels with incident type 2 diabetes, and has shown no association.

Adrenocorticotropic hormone regulates the production of both DHEA and DHEAS, which, once secreted into the bloodstream, are carried bound to albumin. Although DHEAS, the sulphated form of DHEA, can be converted to DHEA, and vice versa, they have some important peculiarities [48]. Concentrations of DHEAS are between 250 and 500 times higher than concentrations of DHEA in women and men, respectively. This difference in concentrations between DHEA and DHEAS depends mainly on the fact that DHEAS is only slowly cleared from the blood, with a clearance rate of 13 l/day, while DHEA is rapidly cleared at a rate of approximately 2000 l/day. Therefore, DHEAS has a half-life of 10–20 h, while the half-life of DHEA is 1–3 h. Diurnal variation of DHEA exhibits a similar pattern to that of cortisol secretion, reaching a peak in the early morning. DHEAS levels are considered not to have a diurnal variation [49].

The strengths of our study include its prospective design, the long follow-up and the comprehensive adjustment for a broad range of possible confounders. Moreover, this is the first population-based study to investigate the associations between the serum levels of DHEA and its main derivatives and incident type 2 diabetes in both men and women. We also performed several sensitivity analyses such as excluding the first 3 years of follow-up to avoid a potential bias of undiagnosed disease at baseline. Furthermore, the diagnosis of incident diabetes was made by standardised blood glucose measurements at the repeated study centre visits and electronic linkage with pharmacy dispensing records in the study area.

However, our study has some limitations. Our population was aged 55 years and older, and therefore results should be generalised to a younger age only with caution. HbA_1c_ and OGTTs were not measured in our study population, which would have strengthened our results. In contrast to DHEAS and its other derivatives, DHEA has a pronounced diurnal rhythm and exhibits a morning elevation similar to cortisol. However, the diurnal secretory pattern of DHEA is more stable and the day-to-day variability less stable than cortisol [50]. Furthermore, this study was carried out in middle-aged and elderly patients, and in older age hormone levels are more stable over time (intraclass correlation 0.75, 0.88 and 0.66 for DHEA, DHEAS and androstenedione respectively) [50]. In addition, the Rotterdam Study mainly includes individuals of European ancestry (98%). Thus, our findings may not be extendable to non-white groups.

We conclude that higher serum levels of DHEA are independently associated with a decreased risk of developing type 2 diabetes in healthy populations of both men and postmenopausal women. These prospective data suggest that DHEA may play a role in the pathogenesis of type 2 diabetes, which may have important implications for preventive interventions.

## Electronic supplementary material

Below is the link to the electronic supplementary material.

ESM (PDF 376 kb)

## Abbreviations

CRP: C-reactive protein
CVD: Cardiovascular disease
DHEA: Dehydroepiandrosterone
DHEAS: Dehydroepiandrosterone sulphate
HDL-C: HDL-cholesterol
LDL-C: LDL-cholesterol
PPAR: Peroxisome proliferator-activated receptor
RCT: Randomised controlled trial
RS-I: Rotterdam Study cohort 1
RS-II: Rotterdam Study cohort 2
SBP: Systolic BP
SHBG: Sex hormone-binding globulin
TC: Total cholesterol
TG: Triacylglycerol
TSH: Thyroid-stimulating hormone
