# Supplementary material for: Serum dehydroepiandrosterone levels are associated with lower risk of type 2 diabetes: the Rotterdam Study
Source: Diabetologia. 2016 Oct 22;60(1):98–106. doi: 10.1007/s00125-016-4136-8 (PMC6518366; doi:10.1007/s00125-016-4136-8)
Supplement: Supplementary file 1 — (PDF 376 kb) [file 125_2016_4136_MOESM1_ESM.pdf]

Electronic Supplementary Material (ESM)

ESM Table 1 Comparison of the predictive value of DHEA with its derivatives.

ESM Fig. 1 Flow chart of subjects eligibility.

ESM Fig. 2 DHEA and its main derivatives in steroidogenesis.

ESM Table 1. Comparison of the predictive value of DHEA with its derivatives.

	c-statistics (95%CI)	c-statistics changes (95% CI) from base model
Base model	0.637 (0.609, 0.666)	NA
+DHEA	0.638 (0.612, 0.665)	0.001 (-0.003, 0.005)
+DHEAS	0.637 (0.610, 0.666)	0.000 (-0.004, 0.005)
+androstenedione	0.637 (0.608, 0.667)	0.000 (-0.002, 0.002)
+DHEAS/DHEA	0.637 (0.609, 0.666)	0.000 (-0.001, 0.001)

Base model: age, sex, parental history of diabetes, body mass index (Wilson`s base model).

ESM Fig. 1.

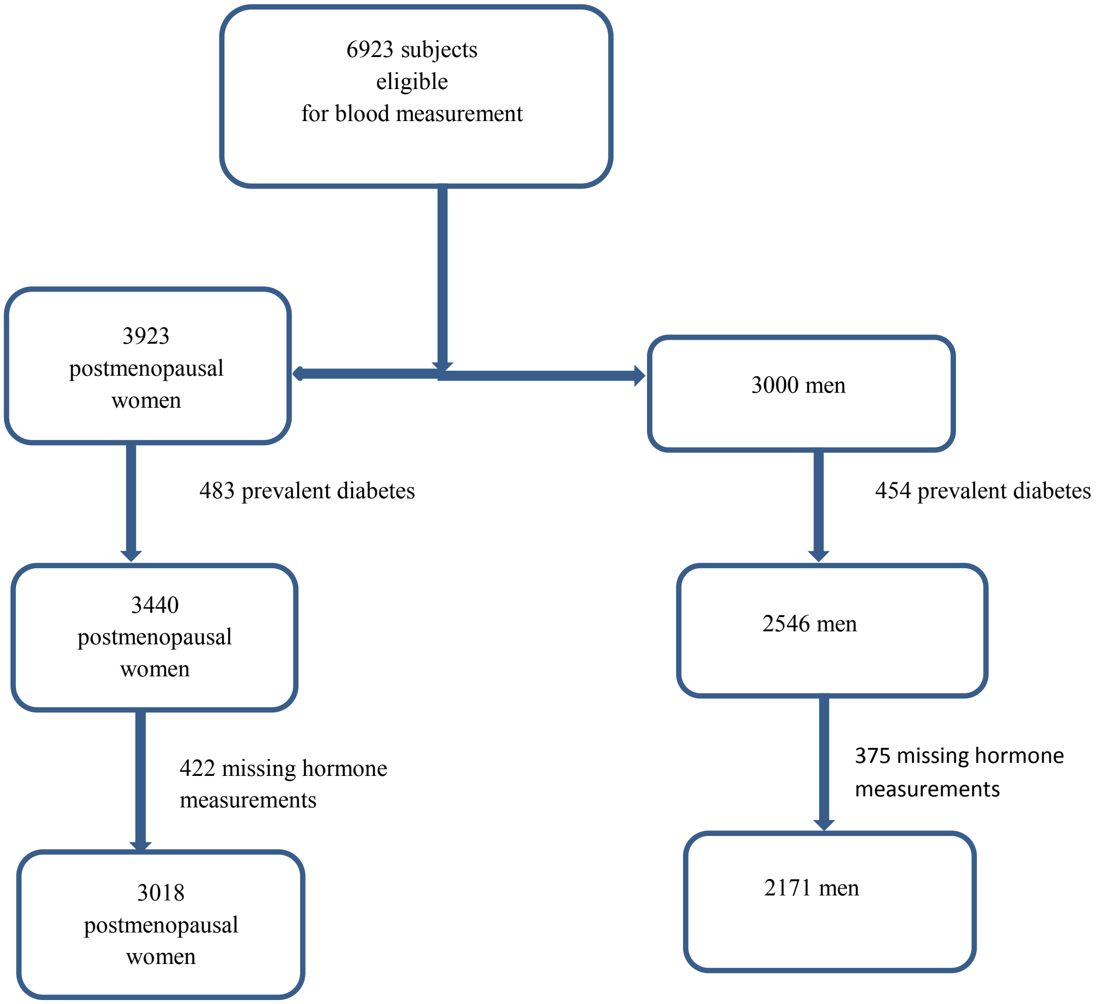

6923 subjects
eligible
for blood measurement
3923
postmenopausal
women
3000 men
483 prevalent diabetes
454 prevalent diabetes
3440
postmenopausal
women
2546 men
422 missing hormone
measurements
375 missing hormone
measurements
3018
postmenopausal
women
2171 men

ESM Fig. 2.

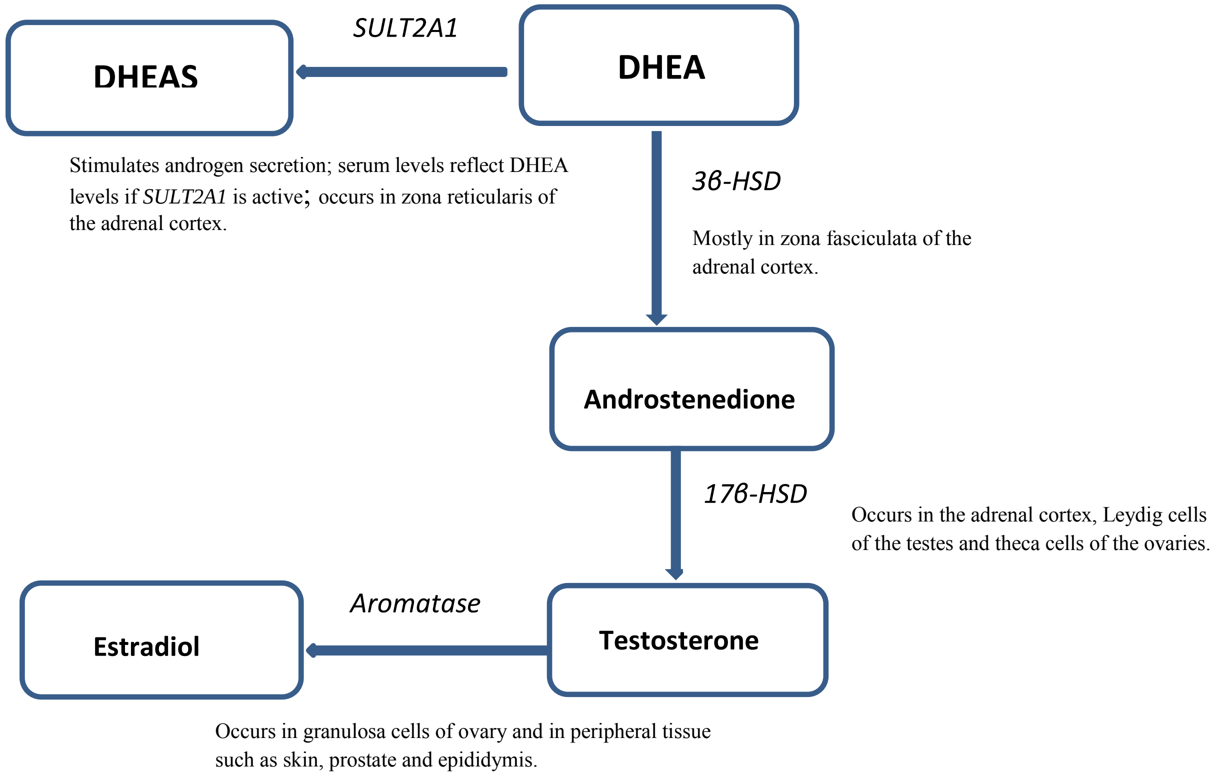
DHEAS
SULT2A1
DHEA
Stimulates androgen secretion; serum levels reflect DHEA levels if *SULT2A1* is active; occurs in zona reticularis of the adrenal cortex.
3β-HSD
Mostly in zona fasciculata of the adrenal cortex.
Androstenedione
17β-HSD
Occurs in the adrenal cortex, Leydig cells of the testes and theca cells of the ovaries.
Estradiol
Aromatase
Testosterone
Occurs in granulosa cells of ovary and in peripheral tissue such as skin, prostate and epididymis.
